# Mandatory Grand Rounds Evaluations: More Data, Less Information

**DOI:** 10.7759/cureus.24567

**Published:** 2022-04-28

**Authors:** Matthew Wecksell, Irim Salik

**Affiliations:** 1 Anesthesia, Westchester Medical Center, New York, USA

**Keywords:** mandatory evaluation, learner evaluation, grand rounds, accreditation, continuing medical education

## Abstract

Aims: For several years, physicians have been required to evaluate a continuing medical education (CME) session before receiving a certificate of participation from an accredited provider. The mandatory nature of these evaluations has led to a high number of evaluations that offer information of questionable utility.

Material and methods: We asked our CME evaluation vendor Eeds for all of the CME evaluation timestamps for our grand rounds from August 5 to September 16, 2020. We obtained time-stamped evaluation data from our CME services vendor and compared the times that sessions were evaluated to the start and completion times of those CME sessions.

Results: While almost all attendees completed electronic evaluations, 8% did so before the start of the session and half did so before its completion.

Conclusions: Making evaluations mandatory has had the effect of lowering the quality of the data thus obtained. In an age that has been described as the “graying of grand rounds,” there are more effective strategies to enhance educational value and learner satisfaction.

## Introduction

According to the Accreditation Committee on Continuing Medical Education (ACCME), “participation in accredited CME helps physicians meet requirements for maintenance of licensure, maintenance of certification, credentialing, membership in professional societies, and other professional privileges ” [1]. Indeed, accredited continuing medical education is now required for medical licensure in 48 states, Puerto Rico, Washington, D.C., the US Virgin Islands, and the Northern Mariana Islands. Obtaining that accreditation requires a CME sponsor to meet several criteria, among them ACCME criteria 11 and 12, which require them to “collect data and information about the changes that result from its educational interventions, including changes it expects learners to make, changes that learners actually make, and/or the impact on patients” and to then use this data in a quality improvement (QI) program [2].

Although it remains an important goal for departments to optimize the educational value of grand rounds conferences, the implementation of a mandatory course evaluation does not serve this purpose. The necessity of obtaining CME means that the required evaluation of the course materials is often seen by learners not as an opportunity to improve the CME process both for them and the sponsor but, rather, as a barrier to obtaining the certificate necessary for the maintenance of licensure, board certification, and other credentialing. Our department offers weekly grand rounds, for which CME is offered when the presentation is of sufficient educational value. Recently, we switched from a paper-based system of collecting CME attendance and evaluation data to an online one. While our CME management vendor does not offer reporting with individual per-evaluation time stamps, they were able to provide us with that information on our request. This article was previously posted to the Research Square preprint server on October 22, 2021.

## Materials and methods

We asked our CME evaluation vendor Eeds for all of the CME evaluation timestamps for our grand rounds from August 5 to September 16, 2020. Eeds is a cloud-based CME integration system that allows organizations to view and track CME credits from each learner’s individual transcript. It also allows participations instantaneous access to course materials, certificates, evaluations, and transcripts. During this time, we had seven grand rounds, five of which offered CME credit. For these five sessions, we had 168 total responses, which is an average of 33.6 responses per session. In our department, 41 would be expected to be available to attend each grand rounds. Our educational sessions run from 6:45 to 7:45 a.m. each Wednesday. For the five weeks we examined, we evaluated the time of day of each response and compared it to the actual start of the didactic session.

## Results

Evaluations of CME grand rounds require the participant to respond to three questions on a five-point Likert scale gauging responses as outstanding, good, average, fair, or poor. Participants are asked to evaluate the content of the presentation, the delivery of information, and the overall presentation. Participants also assess if the speaker was objective and unbiased by commercial interests, the educational relevance of the presentation to clinical practice and professional development, and if and how the activity would affect practice change. Specifically, practitioners are queried as to how the activity will help with improvement in terms of competence or practice performance. Raters are also asked to assess how effective the presentation was in contributing to a change in solving medical problems, the application of new scientific and clinical knowledge, skills, and techniques, perceived self-efficacy in medical ethics and practice, responsiveness to QI issues, and/or the ability to provide safe, efficient and cost-effective healthcare.

Of 168 evaluations, 13 (7.8%) were filled out prior to 6:45 a.m. on the day of the session. An additional 20 (12%) were filled out prior to 7 a.m. Half (n=84) of the evaluations were completed and submitted prior to the conclusion of the didactic session. Of the remainder, 78 were submitted during the workday, with the rest submitted later in the evening. One respondent submitted their CME evaluation at 3:10 a.m. the morning after the session.

## Discussion

Twenty percent of our faculty answered questions relating to the didactic content (i.e. was it free from commercial bias, will it change their current medical practice) before the sessions were a quarter of the way through. Almost 8% answered those questions before the start of the actual session. Only half of our faculty waited until the conclusion of the session before evaluating its content, biases, and its projected effect on their clinical practice. This observed effect is likely due to the physicians’ need to obtain a CME certificate for maintenance of their career (licensure, hospital credentialing), far outweighing their desire to provide meaningful data to the CME providers. Conversation with physicians at other institutions leads the authors to believe that these results, despite being from a small sample at a single institution, are quite generalizable.

In the late 19th century, classic grand rounds were first initiated by Sir William Osler at Johns Hopkins Medical School as a novel method of clinical education [3]. Residents learned through bedside teaching; faculty moved from patient to patient, pontificating on the methodology of disease pathology, diagnosis, and treatment. The traditional model of grand rounds was introduced as teaching transitioned from a patient’s bedside to an auditorium. Classically, patients were present during a resident’s presentation and senior faculty queried the patients and observed physical examination skills demonstrated by the resident. Once the patient left, residents and faculty engaged in “free discussion between thinking men of widely different interests and experience that instilled character and inspired future physicians.”

The mid-to-late 20th century has been described as the “graying of grand rounds,” as patients and their social issues, feelings, and attitudes were no longer the focus. At this time, disease pathology became the most integral discussion topic [4]. This shift in structure to perceived monotonous, mundane, lecture-based didactics has called the relevance of grand rounds into question. There are several reasons for declining grand rounds attendance as well as reduced relevance, prestige, and educational orientation. Potential causes include poor organization, presenters’ poor teaching skills, concomitant clinical responsibilities of attendees, a reduced patient-centered focus, reduced subspecialty practice relevance, reduced participation by departmental leadership, lack of interaction between presenters and attendees, tardiness, food consumption, inconvenient timing and location, and physical limitations of presentation venues.

Literature reviews of CME evaluations have shown that outcome measures, length of follow-up, and evaluation methods all remain variable. In 2005, Curran et al. adapted the 4-level evaluation model by Kirkpatrick for utilization of a summative evaluation of CME [5]. Based on this adapted model, evaluation begins with participant satisfaction (level 1), followed by knowledge acquisition and attitude change (level 2), physician clinical practice change (level 3), and then patient outcomes (level 4). Each level builds on the former, and each successive evaluation represents a more rigorous analysis of overall effectiveness. The optimal CME evaluation would measure the following variables: participant satisfaction, practitioner knowledge and attitude gauged with a validated and reliable outcome measure, participant performance changes in the clinical setting based upon objectively observed data, and finally, improvements in patient outcomes.

Based on the ACCME 2020 data report, most learners transitioned from live courses or regularly scheduled series to online learning activities. Analysis reveals that 98% of accredited education activities are designed to change competence, 55% are designed to change performance, and 28% are designed to change patient outcomes [6]. Educators are tasked with identifying the most effective CME tools to enhance information delivery and bridge the gap between evidence and clinical practice, particularly as these information mediums are utilized for QI initiatives.

Several academic departments attempting to improve the departmental grand rounds experience have investigated attendance patterns, utilized assessment surveys, and developed and implemented a series of subspecialty-specific topics. Presenters have been advised on topic selection instead of leaving it to a lecturer’s discretion. It has been reported that grand rounds are the most expensive conferences in most academic departments, due to stipends for external speakers and the time and opportunity costs for faculty diverted from clinical responsibilities. Despite the aforementioned challenges, most academic medical centers still devote significant time and energy to grand rounds planning, even though its effectiveness is largely unknown. Most academic departments provide CME credit for grand rounds attendance, and one study found that half of the faculty primarily used grand rounds attendance for CME credit [7].

## Conclusions

Allowing participants in a CME session to provide feedback and evaluation of the session may generate useful data. Requiring evaluation will generate a significant amount of excess data, much of it before the conclusion, or even the start of the educational session. Treating adult learners as captive data collection participants in a quality improvement project does a disservice both to those learners and to the CME content providers who receive feedback that is unrelated to their performance as teachers or the quality of their didactic. Making evaluations mandatory has had the effect of lowering the quality of the data thus obtained. The ACCME criteria 11 and 12 are quite high-minded, but in practice, only serve to create the appearance of addressing knowledge/performance gaps while serving as a mere obstacle between physicians and the CME hours they need to accumulate to maintain licensure. For academic departments looking to improve grand rounds quality, there are more effective ways to do so.

## References

[1] (2020). Why accredited CME matters. https://www.accme.org/why-accredited-CME-matters.

[2] (2020). Accreditation criteria. https://www.accme.org/accreditation-rules/accreditation-criteria.

[3] Beeson PB (1986). One hundred years of American internal medicine. A view from the inside. Ann Intern Med.

[4] Ingelfinger FJ (1978). Sounding boards. The graying of grand rounds. N Engl J Med.

[5] Johnston S, Coyer FM, Nash R (2018). Kirkpatrick's evaluation of simulation and debriefing in health care education: a systematic review. J Nurs Educ.

[6] Hebert RS, Wright SM (2003). Re-examining the value of medical grand rounds. Acad Med.

[7] Mueller PS, Litin SC, Sowden ML, Habermann TM, LaRusso NF (2003). Strategies for improving attendance at medical grand rounds at an academic medical center. Mayo Clin Proc.

